# Effect of black tea extract on fresh rice noodles: multiple quality attributes and underlying mechanism

**DOI:** 10.1016/j.fochx.2025.103007

**Published:** 2025-09-14

**Authors:** Tong Chen, Rongrong Xu, Xiaohui Gao, Shuyu Wei, Qianwei Cheng, Luli Meng, Jiayan Zhang, Yuan Cheng

**Affiliations:** a School of Biological and Chemical Engineering, Guangxi University of Science and Technology, Guangxi Liuzhou Luosifen Center of Technology Innovation, Liuzhou 545006, Guangxi, China.; b Liuzhou Quality Inspection and Testing Research Center, Liuzhou 545001, PR China.; c Dalian University of Finance and Economics, School of Accounting, Dalian 116622, PR China.

**Keywords:** Fresh rice noodles, Black tea extract, Multivariate statistics, Quality, Microstructure

## Abstract

Fresh rice noodles (FRNs) are nutritionally simple and prone to aging, which limits their shelf life and quality. This study investigated the effects of black tea water extract (BTWE) at various tea:water ratios (0:100 (control), 4:100, 6:100, 8:100, and 10:100) on the overall quality of FRNs and explored the underlying microstructures and molecular mechanisms. The results showed that BTWE significantly enhanced textural properties, antioxidant capacity, and flavor, while reducing breakage rate without affecting cooking loss. A tea:water ratio of 8:100 marked a key transition point in quality differentiation. Color shifts toward red and yellow were observed with increasing BTWE. Key volatile compounds, including nonanal, n-hexadecanoic acid and linoleic acid, contributed to flavor differences. Microscopic analyses revealed that polyphenols in black tea promoted protein-starch aggregation, forming a compact gel network, while spectroscopy confirmed hydrogen bond formation between polyphenols and starch. These findings highlight BTWE's potential as a natural additive to enhance the comprehensive quality of FRNs.

## Highlights

• BTWE enhanced multiple quality attributes of FRNs, including texture, color, antioxidant capacity, and flavor.
• Key flavor compounds (Nonanal, n-Hexadecanoic acid, Linoleic acid, and Caffeine) were identified in FRNs with BTWE.
• Low concentration of tea polyphenols enhanced gel stability by promoting protein-starch aggregation and hydrogen bonding.
• High concentration of tea polyphenols reduced gel stability by competing with starch molecules for hydrogen bonding.
• Optimal quality was achieved in FRNs at a tea:water ratio of 8:100.

## 1 Introduction

Rice is a key starch-based staple crop globally. Rice contains essential amino acids, lipids, and carbohydrates required for human nutrition and provides ∼21 % of the calories required by the human body (Huang et al., 2022). Rice noodles are made primarily from rice and can be round or flat in shape. They are produced through a series of procedures, including soaking, crushing (or grinding), ripening, extruding, and molding (or cutting) (Li, You, et al., 2021), and are important staple foods in the southern and Southeast Asian regions of China. Currently, rice noodles can be divided into fresh, semi-dried, and dried products according to their moisture content (Xiao et al., 2022). Fresh rice noodles (FRNs), with the advantages of rich flavor and easy steaming and cooking, are appreciated by consumers more than other rice noodles. However, it is also prone to aging and deterioration during the storage, and cannot meet the diverse needs of consumers. Changes in physical and chemical properties of starch, the main nutrient in rice noodles, significantly impact their quality (Chen et al., 2022). However, rice noodles with a low protein content cannot easily form a uniform and cohesive texture, which is prone to higher losses and easy breaking during the cooking process (Kasunmala et al., 2020). Therefore, further research is needed on natural improvers to enhance the original quality of FRNs.

Black tea is rich in tea polyphenols, amino acids, minerals, and other bioactive components. It has a strong aroma, fresh flavor, and potent antioxidant properties. Black tea also holds great potential for preventing or reducing the risk of diabetes, cardiovascular diseases, and cancer (Shaukat et al., 2023). These health-promoting attributes are particularly valuable for the development of tea-based foods. Recently, the use of tea as a novel food improver to diversify staple foods (like Chinese noodles, Chinese steamed buns, and cakes) has become a popular research topic. As a novel food additive, tea is typically incorporated into foods in the form of tea extracts, tea powder, tea polyphenols, or tea polysaccharides to develop functional, tea-based products (Zhang et al., 2015). This shift from “drinking tea” to “eating tea” can not only make full use of beneficial tea components but also enhance product quality in a healthy and safe manner (Wang et al., 2023). The addition of mulberry leaf tea extract could enhance the nutritional value and antioxidant capacity of rice noodles while also improving their textural properties during storage (Makchuay et al., 2023). Oolong tea soup has also been reported to enhance the quality of rice noodles, resulting in a novel product with a unique flavor and strong antioxidant activity (Huang, Lai, et al., 2024). In addition to enhancing the flavor and quality of rice noodles, tea polyphenols (e.g., catechins) have been shown to effectively delay noodle quality deterioration due to their excellent antioxidant properties (Li et al., 2020; Ping et al., 2025). These compounds are considered key active ingredients contributing to the extension of product shelf life. However, the texture characteristics of rice noodles containing tea extract still require improvement. It is due to that high content of tea polyphenols can interact with starch molecules, thereby inhibiting the formation of ordered structures and crystallization during starch retrogradation. This interaction weakens the structural integrity of the starch gel network, ultimately resulting in reduced hardness and cohesiveness of rice noodles (Huang, Li, et al., 2024). Furthermore, tea polyphenols can change the starch structure through hydrogen bonding, delay starch digestion (Li, Xiao, et al., 2021), and significantly inhibit starch retrogradation (Lv, Li, et al., 2020a; Lv, Li, et al., 2020b). In summary, existing studies mainly focus on the development of rice noodles with special flavors or functional properties, while few reports have explored the development of FRNs and the mechanism by which tea compounds improve the quality of rice noodles. Therefore, exploring the extraction and application of beneficial components from tea in FRNs production is worthwhile, aiming to develop high-quality, naturally healthy and eco-friendly rice products.

The study investigated the effect of black tea at different tea infusion ratios (0:100 (control), 4:100, 6:100, 8:100, and 10:100) on the quality properties (cooking loss, breakage rate, texture, color, antioxidant properties, and flavor) and microstructure of FRNs. Multiple techniques, including confocal laser scanning microscopy (CLSM), scanning electron microscopy (SEM), X-ray diffraction (XRD) and Fourier transform infrared spectroscopy (FTIR) were used to analyze the changes in the internal microscopic image and molecular structure of FRNs treated with black tea water extract (BTWE) to elucidate the mechanism of its influence.

## 2 Materials and methods

### 2.1 Materials

Indica rice (Fuxiang brand) was provided by Fuxiang Grain and Oil Co., Ltd. (Guangxi, China). Black tea (semi-fermented tea) was purchased from Liuzhou Sanjiang County Youhe Tea Co., Ltd. (Guangxi, China). Sodium chloride and anhydrous ethanol were purchased from Xilong Science Co., Ltd. (Sichuan, China). 2,2-Diphenyl-1-picrylhydrazyl (DPPH) antioxidant assay kit was purchased from McLean Biochemical Technology Co., Ltd. (Shanghai, China). Fluorescein 5-isothiocyanate (FITC) and Rhodamine B were provided by Fuzhou Feijing Biotechnology Co., Ltd. (Fujian, China). Potassium bromide was purchased from Guangfu Science and Technology Development Co., Ltd. (Tianjin, China).

### 2.2 Black tea water extract preparation

We followed a published method (Yu et al., 2019) with slight modification. Specifically, a mill (Wenling Lin Da Machinery Co., Ltd., Wenling, China) was used to grind black tea into a powder, which was passed through the 100-mesh sieve, and then the powder was extracted under a water bath at 95 °C for 10 min in different tea:water ratios (4:100, 6:100, 8:100, and 10:100), respectively. After cooling to room temperature, the mixture was centrifuged at 4000 rpm in a centrifuge (Jintan Medical Instrument Factory, Jintan, China) for 10 min, then the supernatant was transferred to a 100 mL volumetric flask and fixed with distilled water to obtain the BTWE, which was stored at 4 °C for later use.

### 2.3 Fresh rice noodles preparation

Indica rice samples were ground into a powder in a mill (Wenling Lin Da Machinery Co., Ltd., Wenling, China) and passed through a 100-mesh sieve. Next, 60 mL BTWE solution was mixed with 100 g rice flour, and then steeped and fermented at 40 °C for 2 h. After soaking and fermenting process, the formed rice paste was steamed at 100 °C for 6 min, and then kneaded to form a ball and subsequently extruded into round, bar-shaped rice noodles (diameter: 0.4 mm) by the extruder, which were eventually rinsed with cold water after secondary maturation and drained as FRNs. Rice noodles soaked in distilled water served as the control group.

### 2.4 Quality properties

#### 2.4.1 Cooking loss

FRNs (5 g) were placed in 500 mL of boiling distilled water for 3 min, after which the FRNs were removed and their surfaces were rinsed with 50 mL of distilled water. Then, the cooking water and rinsing water were transferred to an oven at 105 °C for evaporation to a constant weight. Cooking loss was expressed as the percentage of solids lost during cooking. Cooking loss (%) was calculated using Eq. (1).

(1) $$\mathrm{Cooking loss}\left(\%\right)=\frac{\mathrm{Weight of dried residue in cooking and rinse water}}{\mathrm{Weight of fresh and}\;\mathrm{wet}\;\mathrm{rice noodles}}\times 100$$

#### 2.4.2 Breakage rate

100 g of FRNs with a length of 20 cm or more were selected and placed in 500 mL boiling water for 3 min, removed, and drained in cold water. Then, FRNs shorter than 10 cm were selected and weighed (denoted as *M*_*1*_), FRNs longer than 10 cm were weighed (denoted as *M*_*2*_), and the breakage rate (*L*) was calculated according to Eq. (2).

(2) $$L\left(\%\right)=\frac{M_{1}}{M_{1}+M_{2}}\times 100$$

#### 2.4.3 Texture characteristic

The texture characteristics (hardness, springiness, chewability, and cohesiveness) of FRNs were measured using a CT3–100 TPA Texture Analyzer (AMETEK Brookfield, USA), equipped with a TA4/1000 cylindrical probe. The TPA mode was selected, the test speed was set to 0.5 mm/s, the trigger force was set to 5 g, and the detection distance was set to 1 mm.

#### 2.4.4 Color characteristic

The color (L*, a*, and b*) characteristics of each FRN were determined using a LS171 colorimeter (Shenzhen Linshang Technology Co., Ltd., China), where L* represents brightness, a* indicates red-green value, and b* indicates blue-yellow value. Whiteness (*WI*) was calculated according to Eq. (3), and total color difference (Δ*E*) was determined using Eq. (4).

(3) $$\mathrm{WI}=100-\sqrt{{\left(100-L^{*}\right)}^{2}+{\left(a^{*}-a_{0}\right)}^{2}+{\left(b^{*}-b_{0}\right)}^{2}}$$

(4) $$\Delta E=\sqrt{{\left(L^{*}-L_{0}\right)}^{2}+{\left(a^{*}-a_{0}\right)}^{2}+{\left(b^{*}-b_{0}\right)}^{2}}$$

#### 2.4.5 Antioxidant properties

DPPH radical scavenging activity was measured using a modified version of an existing method (Fu et al., 2021). First, FRNs were sieved through a 100-mesh screened after freeze-drying process. Then, 0.5 g of the FRNs powder was mixed with 20 mL of 75 % anhydrous ethanol in a 50 mL centrifuge tube and sonicated at 50 °C for 1.5 h. Subsequently, the mixture was centrifuged at 3000 rpm for 10 min, and the supernatant was collected for further use. After that, 2 mL of 0.20 mM DPPH solution was added to a 0.5 mL sample solution and allowed to react for 30 min at room temperature, protected from light. The absorbance value (*A*_*s*_) was measured at 517 nm. Additionally, following the same procedure as described above, 2 mL of anhydrous ethanol was added to a 0.5 mL sample solution, and then measured to obtain the absorbance value (*A*_*b*_). Meanwhile, 2 mL 0.20 mM DPPH solution was added to 0.5 mL of anhydrous ethanol, and then measured to obtain the absorbance value (*A*_*0*_). The DPPH radical scavenging activity was calculated according to Eq. (5).

(5) $$\mathrm{DPPH radical scavenging activity}\left(\%\right)=\left(1-\frac{A_{S}-A_{b}}{A_{0}}\right)\times 100$$

#### 2.4.6 Flavor characteristic

Volatile organic compounds (VOCs) in each FRNs were analyzed using GC (8890D, Agilent Technologies, Inc., USA) combined with MS (7000D, Agilent Technologies, Inc., USA), which was also equipped with an autosampler (CTC Analytics AG, Zwingen, Switzerland). The specific operations were: 5 g FRNs and 4 mL saturated sodium chloride solution were added into a 20 mL headspace vial, 2-octanol (336 μg/L) was added as an internal standard, then the vial was heated to equilibrate at 90 °C for 30 min. After that, an arrow-shaped solid-phase micro-extraction head (DVB/Carbon-WR/PDMS, 1.10 mm, 120 μm) was inserted into the headspace vial for constant-temperature extraction and adsorption (lasted for 40 min), and then the extraction head was desorbed in the inlet port at the temperature of 250 °C for 5 min. Notably, the extraction needle was aged in the GC injection port at 250 °C for 3 min before use. Finally, the instrument was activated to collect VOCs data.

The GC conditions: The instrument was equipped with an HP-5MS column (30 m × 250 μm × 0.25 μm). Helium (He) was used as the carrier gas, with a flow rate of 1 mL/min, a split ratio of 5:1. The column temperature program was as follows: The initial column temperature was set at 40 °C during the initial 3 min, then increased to 165 °C at a rate of 5 °C/min and held for 5 min, next raised to 250 °C at a rate of 4 °C/min, followed by the final stage for 5 min.

The MS conditions: EI ionization was applied at 70 eV and the temperature was set at 230 °C; the transmission line temperature was 280 °C, and the quadrupole temperature was 150 °C; the full mass scanning range was 32–500 *m*/*z*.

Qualitative and quantitative analyses: Compounds were identified by searching the NIST 17 database (National Institute of Standards and Technology), filtered with a match factor ≥ 80 %; the relative concentration of each volatile flavor compound in the sample was determined based on the amount of internal standard added.

### 2.5 Scanning electron microscopy

FRNs were freeze-dried, cut into thin slices, and their cross-sections were sprayed with gold and then transferred to a SEM (Apreo 2C, Thermo Fisher, USA) to observe the microstructure of FRNs at 1000 × magnification.

### 2.6 Confocal laser scanning microscope

FRNs were cut into thin slices and placed on slides that were stained with 0.2 % FITC solution and 0.025 % rhodamine B solution for 10 min. The samples were then rinsed with a small amount of distilled water and wiped with filter paper. Microscopic images were obtained under a CLSM (A1R, Nikon Corporation, Japan) at a resolution of 1024 × 1024 pixels. The excitation wavelengths for FITC and rhodamine B were 488 and 561 nm, respectively.

### 2.7 X-ray diffraction spectroscopy

Each sample was freeze-dried and pulverised through a 100-mesh sieve, and XRD patterns of each sample were obtained using an XRD (D/MAX-3BX, Rigaku Holdings Corporation, Japan). The 2θ scanning range was set at 4–40°, scanning rate was 2°/min, and scanning step was 0.02°.

### 2.8 Fourier-transform infrared spectroscopy

FRNs were sieved through a 100-mesh screen after the freeze-drying process, and then the sample powder was mixed with potassium bromide at a ratio of 1:100 (*w*/w), followed by grinding and pressing. Infrared spectra were recorded using a Fourier transform infrared spectrometer (FTIR, Frontier/Nicolet 380, PerkinElmer, USA) in the range of 4000–400 cm^−1^. The scanning resolution was set at 4 cm^−1^ and the scanning time was set at 32.

### 2.9 Statistical analysis

One-way analysis of variance (ANOVA) was performed using SPSS 19.0 software (IBM, Chicago, USA). FTIR spectra were processed and analyzed using OMNIC 8.2 software, while principal component analysis (PCA) and clustering analysis were conducted using MATLAB R2019a software (MathWorks, Inc., USA). Amide *I* band were fitted using PeakFit 4.12 software (Systat Software, Inc., USA). Graphs were drawn using the Origin 9.0 software (OriginLab Corporation, USA). Each measurement was repeated three times for all assays and the average value was used to express the final result.

## 3 Results and discussion

### 3.1 Quality characteristic analysis

#### 3.1.1 Cooking quality

**Fig. 1:**
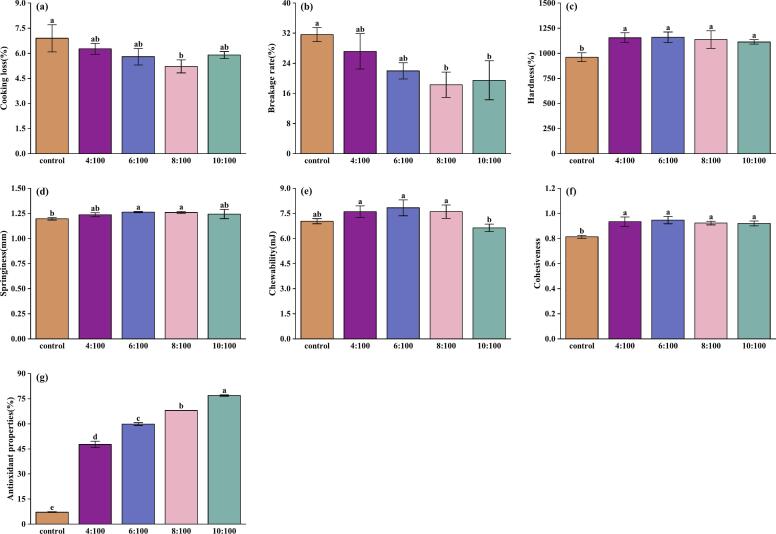
Black tea water extract effect on multiple quality attributes of fresh rice noodles. (a) Cooking loss; (b) Breakage rate; (c) Hardness; (d) Springiness; (e) Chewability; (f) Cohesiveness; (g) Antioxidant properties. Different letters within column indicate significant differences in means (*p* <0.05).

Cooking quality is an evaluation standard of product quality for consumer consumption, and its external performance includes the turbidity of rice soup and the degree of breakage of rice noodles after cooking (Bae et al., 2019). The effects of BTWE on the cooking characteristics of FRNs are shown in Fig. 1(a) and Fig. 1(b). Breakage rate was significantly reduced in FRNs containing BTWE compared to those in the control group. After the tea:water ratio reached 8:100, no significant differences were observed between adjacent ratio samples. Although the cooking loss decreased with the increase of tea:water ratios in FRNs, the overall differences were not significant. This phenomenon might be due to the presence of tea polyphenols in BTWE, which enhanced cross-linking with starch and proteins to form a stable three-dimensional network structure (Yu et al., 2019), making the gel network structure denser inside the FRNs (Song & Yoo, 2017). Therefore, FRNs containing BTWE are less prone to breakage and cooking loss could also be reduced during the cooking process (Li, Xiao, et al., 2021).

#### 3.1.2 Textural properties

The effects of BTWE on the textural properties of FRNs are shown in Fig. 1(c) to Fig. 1(f). Compared to the control group, the addition of BTWE increased several textural parameters of FRNs, including hardness, elasticity, and cohesiveness, except for chewiness. Good elasticity could impart a smooth and pleasant texture to the rice noodles, while higher hardness could contribute to chewiness and enhance the overall quality (Zhang et al., 2021). Although chewiness parameter increased with the BTWE concentration, the difference was not significant. However, when the tea:water ratio reached 10:100, the chewiness decreased instead. Furthermore, the increase in textural parameters might also represent improved cooking resistance, and indicated that the newly formed internal network structure of FRNs was less prone to be disrupted.

#### 3.1.3 Antioxidant activity

Fig. 1(g) shows that the free radical scavenging activity of FRNs containing BTWE was significantly higher than that of the control group, and it gradually increased with the tea:water ratio, which is consistent with previous reported findings (Huang, Li, et al., 2024). This is because BTWE contains abundant polyphenolic compounds, which are rich in O—H groups, the functional groups responsible for scavenging oxygen free radicals. Furthermore, these polyphenols exhibited reductive properties. When cells are threatened by free radicals, they undergo oxidation to form more stable compounds, thereby protecting cellular components from damage and contributing to human health (Fu et al., 2021; Guo et al., 2022).

#### 3.1.4 Color characteristics

**Fig. 2:**
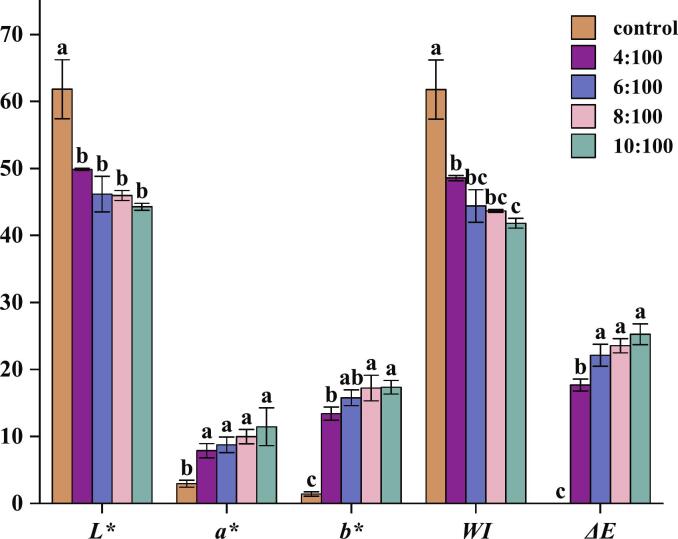
Black tea water extract effect on the color characteristic of fresh rice noodles. Different letters within column indicate significant differences in means (*p* <0.05).

The color changes of all FRNs are shown in Fig. 2. Compared to the control group, FRNs treated with BTWE had significantly lower L* values and higher a* and b* values, which indicated that BTWE significantly reduced the transparency and changed the original color of FRNs. Furthermore, BTWE reduced the saturation level of FRNs, resulting in a lower *WI* and a higher Δ*E*. However, when the tea:water ratio was increased to 6:100, *WI* and Δ*E* did not change significantly, indicating that the color change of FRNs was limited when the tea:water ratio was increased to a certain level. These color changes in FRNs were primarily attributed to the oxidation of polyphenols in black tea, which could darken the color of FRNs and provide consumers with a unique sensory experience (Zhu et al., 2016).

### 3.2 Cluster analysis

**Fig. 3:**
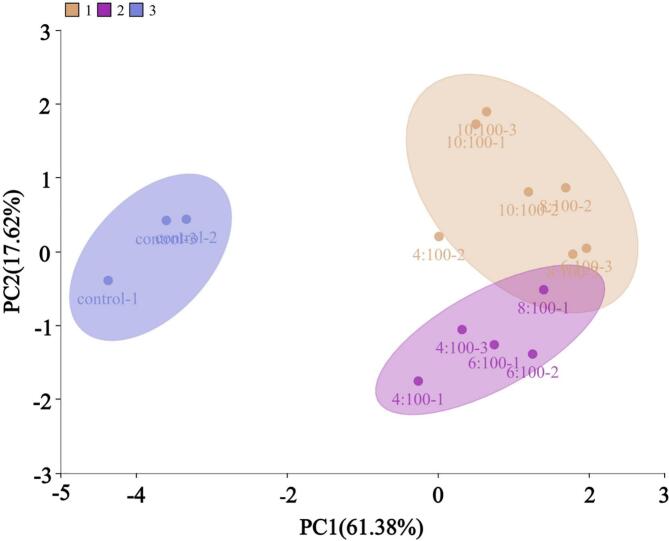
K-nearest neighbor cluster result of multiple characteristics of fresh rice noodles.

To further analyze the impact of BTWE addition on the overall quality of FRNs, the K-nearest neighbor algorithm was used for cluster analysis after normalizing multiple quality data. The results were shown in Fig. 3. As observed, the FRNs were divided into three distinct regions, and the control group was clearly separated from other samples containing BTWE. This indicated that BTWE addition could significantly alter the original quality of FRNs. Notably, the samples with a tea:water ratio of 8:100 were located at the intersection of the other two cluster regions, suggesting that as BTWE concentration increased, a turning point in the quality changes of FRNs emerged. Moreover, the appropriate concentration of BTWE could contribute to an overall improvement in the inherent quality of FRNs.

### 3.3 Flavor analysis

#### 3.3.1 Flavor composition change

**Fig. 4:**
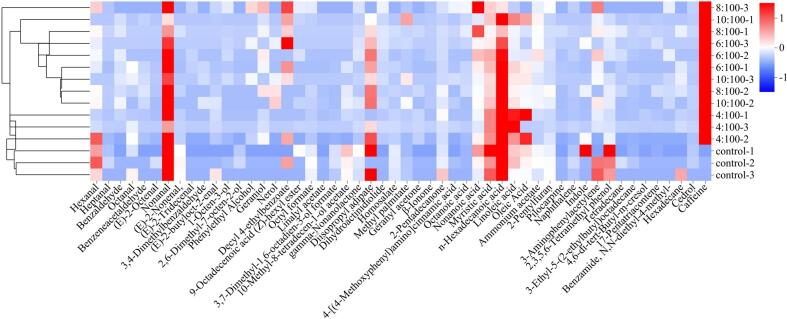
Heat map of relative content of volatile organic compounds in fresh rice noodles.

For flavor analysis, FRNs containing BTWE at different tea:water ratios were analyzed by GC–MS, and the results are shown in Fig. 4. As shown, a total of 52 volatile compounds were identified, primarily including aldehydes, alcohols, ketones, and acids, with most of these compounds aligning with the known aroma components of black tea (Lu et al., 2024). The variety of aldehydes in FRNs increased with the BTWE addition. For example, benzaldehyde has a nutty almond aroma and benzeneacetaldehyde has a honey-like sweet flavor, and they have also been found and proven to significantly impact sweetness in existing research (Kang et al., 2019). Moreover, the type and relative content of alcohols in FRNs with BTWE were higher than those in the control group, which has been confirmed as the primary aromatic component of black tea. Therefore, the addition of BTWE enhances the aroma profile of FRNs (Ma et al., 2022). Notably, all samples contained 1-octen-3-ol, a compound with a characteristic mushroom aroma (Zhang et al., 2021). The increased level of this compound has been shown to contribute to the taste and flavor of rice noodles (Li et al., 2025). Furthermore, the addition of BTWE generated geraniol and nerolidol, which impart citrus and floral scents to FRNs (Kraujalyte et al., 2016). In addition, geraniol has been identified as the key compound of the sweet aromatic in black tea (Wei et al., 2025). In contrast, the addition of BTWE generated several new ketones in FRNs. Among them, β-violet ketone had been identified as an important active odorant that could provide complex fruity and woody flavors (Du et al., 2014). β-violet ketone, primarily formed through enzymatic, oxidative, and cleavage degradation of carotenoids, is the main contributor to the floral aroma in tea infusions (Wu, Chen, et al., 2022). Seven acids were also detected, which contributed less to the flavor of FRNs. However, some acids such as linoleic acid can be further converted into aldehydes through an oxidation reaction (Ge et al., 2022). Additionally, indole is an important aroma-presenting substance that can provide a floral aroma (Zeng et al., 2016) and caffeine can provide the bitter flavor of black tea. These compounds collectively enhance the flavor quality of FRNs. Thus, BTWE can redistribute the volatile flavor substances in FRNs, imparting them a unique flavor.

#### 3.3.2 PCA analysis

**Fig. 5:**
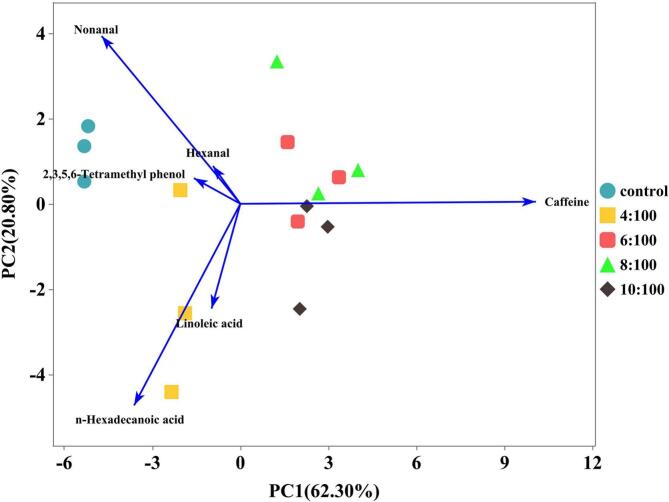
Principal component analysis loadings chart and scores chart of volatile compounds in fresh rice noodles.

To further explore the differences in VOCs among FRNs, the content of each flavor substance (as a variable) was collected and subjected to PCA. The score and loading plots are shown in Fig. 5. After PCA transformation, the cumulative variance contribution of the first two principal components was 83.10 %, indicating that the original data could still effectively maintain the most flavor component information of FRNs after the dimensionality reduction process. Under the new coordinate system, a greater distance between samples indicated a greater difference in sample flavor quality. As shown, the differences in flavor quality between the control group and other FRNs containing BTWE were significant. However, when the ratio of tea:water ratio reached 6:100, there was an overlap between the samples as the ratio of tea:water increased, which suggested a partial similarity in odor and fewer differences in flavor quality. This implied that when the ratio of tea:water reached a certain level, further increase in tea extract content had a limited effect on the overall flavor of FRNs, which was consistent with the previous analysis of physical and chemical properties of FRNs.

The importance of variables in the PCA loadings was measured by the distance between the point corresponding to each volatile flavor compound in the loading plot and the origin of the axis. A greater distance indicated a greater contribution of the volatile flavor compound to the overall flavor differentiation. Due to the large number of feature variables, only the first six critical VOCs variables were selected for analysis. As shown in Fig. 5, the flavor substances, including nonanal, n-hexadecanoic acid, and linoleic acid were located in the left same quadrant as the control group and the samples with a tea:water ratio of 4:100, indicating that these compounds can be used as a typical component to achieve differentiation from other FRNs. The smaller the angle between different variables, the stronger the correlation between them. For example, the angle between the the hexanal and nonanal variables was relatively small, indicating a high correlation between them. Nonanal could distinguish FRNs with the tea:water ratio of 6:100 from other FRNs. Caffeine could be considered as the key compound for distinguishing FRNs with the tea:water ratio of 10:100 from other FRNs. Linoleic acid, 2,3,5,6-tetramethylphenol and hexanal were closer to the origin, indicating a lower contribution to the flavor of FRNs with tea:water ratios below 6:100. All these components could be used as key variables to further distinguish FRNs with different ratios of tea:water. In summary, as the tea:water ratio increased, the overall flavor of FRNs changed with the increasing concentration of BTWE. The characteristic volatile compounds varied at different ratio stages, which could be beneficial for the future development of specialty rice noodles with distinct aromas.

### 3.4 Microstructure analysis

#### 3.4.1 SEM analysis

**Fig. 6:**
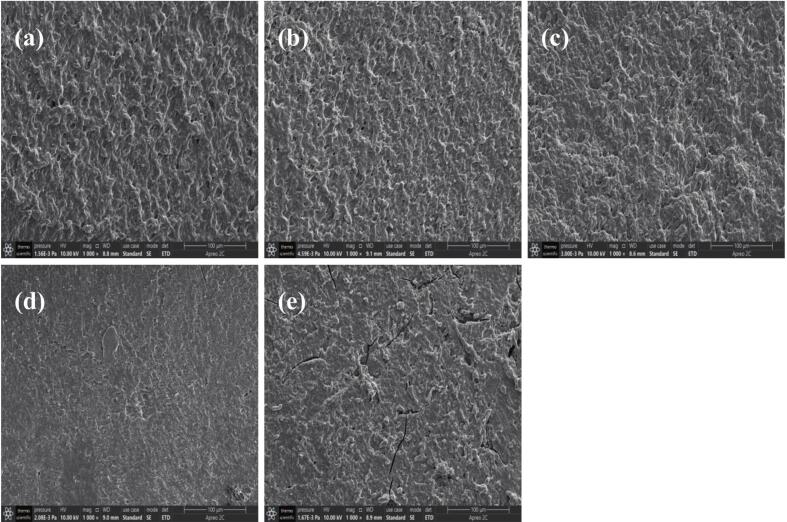
Scanning electron microscopy micrographs of fresh rice noodles. (a) The control group; (b) The tea:water ratio of 4:100; (c) The tea:water ratio of 6:100; (d) The tea:water ratio of 8:100; (e) The tea:water ratio of 10:100.

The microstructural morphology of each sample was observed using SEM. As shown in Fig. 6, FRNs from the control group (Fig. 6(a)) showed a looser gel network structure with larger voids, whereas FRNs containing BTWE had smaller holes with a homogeneous and dense structure, which might be attributed to the presence of a three-dimensional network structure formed by polyphenols, starch, and proteins, resulting in the formation of denser pores and solid gel structures (Li, Xiao, et al., 2021; Srikaeo et al., 2018). This network structure helped retain starch particles during cooking, thereby reducing the cooking loss and breakage rate of FRNs, and resulting in greater hardness, elasticity, and chewiness. However, when the ratio of tea:water was high (see Fig. 6(e)), the network structure was destabilized, and these network holes started to become larger and damaged compared with those in Fig. 6(c) and Fig. 6(d). These larger pores facilitated moisture penetration during steaming, allowing uncoated starch granules within the rice noodles to leach into the cooking soup. This led to higher cooking loss and breakage rate of FRNs, along with reduced hardness, elasticity and chewiness. This structural change also verified the previous speculation about the decrease in texture characteristics at a tea:water ratio of 10:100, where the FRNs were severely eroded by water molecules owing to the inner loose gel structure and larger pore size during the cooking process, finally leading to a decrease in texture properties.

#### 3.4.2 CLSM analysis

**Fig. 7:**
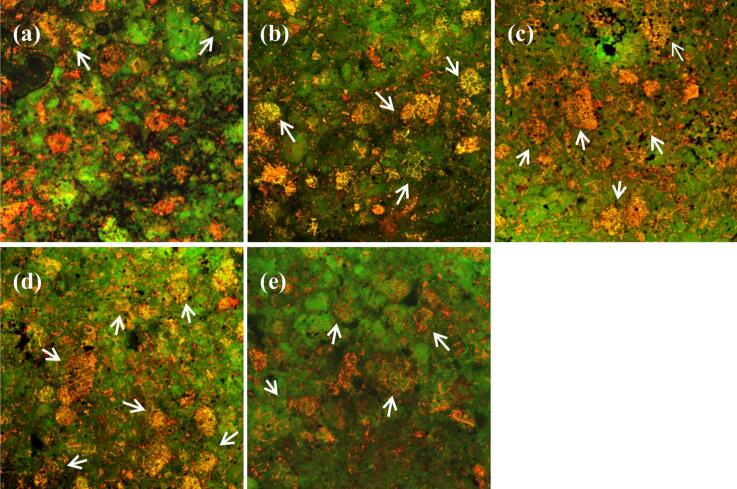
Confocal laser scanning microscopy images of fresh rice noodles. (a) The control group; (b) The tea:water ratio of 4:100; (c) The tea:water ratio of 6:100; (d) The tea:water ratio of 8:100; (e) The tea:water ratio of 10:100.

CLSM was used to observe the internal structure of FRNs (Fig. 7). As shown, starch was marked green by FITC, protein was labeled red by Rhodamine B, and the yellow region represented the composite structure of starch and protein (Jia et al., 2019). The control group (Fig. 7(a)) showed more and incomplete red network areas with fewer yellow regions, indicating protein distribution was scattered and network structure was incomplete. With the addition of BTWE, there was an increased trend in the yellow area in the image (see Fig. 7(b) to Fig. 7(e)), and an expandable and well-developed reticulation gradually appeared, which implied the enhancement of the entanglement formed in the aggregation network between protein and starch molecules (Jia et al., 2020), and the formation of a well-developed three-dimensional network structure facilitated by the polyphenols. Fig. 7(e) showed a slight decrease in the yellow areas compared to Fig. 7(c) and Fig. 7(d), which was consistent with the previous SEM observations. The reason may be that the addition of excessive BTWE possibly disrupted the equilibrium structure of the three-dimensional network.

### 3.5 Functional group analysis

#### 3.5.1 XRD spectroscopy analysis

There were two types of interaction forces between polyphenols and starch. The first is the embedding of polyphenol molecules in the cavity of the starch helical lumen through hydrophobic interactions to form a V-type inclusion complex. The other is a non-inclusion complex in which polyphenols can form a complex with starch molecules through hydrogen bonding or electrostatic interactions (Deng et al., 2023; Liu et al., 2024).

**Fig. 8:**
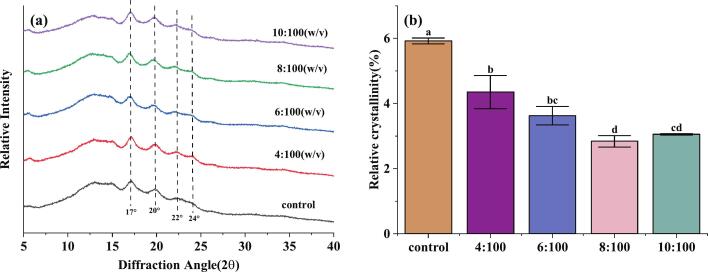
Black tea water extract effect on the X-ray diffraction patterns of fresh rice noodles. (a) Crystal structure; (b) Relative crystallinity. Different letters within column indicate significant differences in means (*p* <0.05).

XRD is an effective tool for analyzing the crystal structure of starch granules and for characterizing the long-range ordered structure of starch. The X-ray diffraction patterns of the FRNs with different tea:water ratios are shown in Fig. 8(a). As shown, all FRNs showed B and V types (17°, 20°, 22°, and 24°) diffraction patterns, and no new diffraction peaks were generated, indicating that BTWE addition did not change the crystal type of starch (Li, Zhai, et al., 2021). Compared to the A-type crystal structure of natural rice starch, the B-type crystal starch in the samples predominantly existed in an amorphous, clustered form and contained more interhelical water molecules, which could promote the formation of a more stable hydrogen bonding network (Wu, Qiao, et al., 2022). As shown in Fig. 8(b), the degree of crystallinity decreased compared to the control group, suggesting that tea polyphenols in BTWE could interact with starch to form hydrogen bonds, which would significantly inhibit the re-association and re-formation of crystalline domains of free starch chains (Du et al., 2019), interfering with the formation of a long-range ordered structure of starch and preventing recrystallisation, thus reducing crystallinity (Liu et al., 2017).

#### 3.5.2 FTIR spectroscopy analysis

**Fig. 9:**
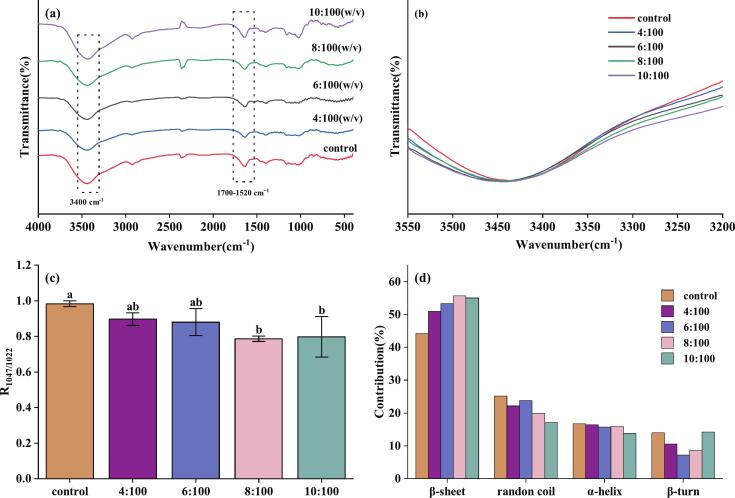
Black tea water extract effect on Fourier transform infrared spectroscopy spectra of fresh rice noodles. (a) 4000–400 cm^−1^; (b) Around 3400 cm^−1^; (c) R_1047/1022_ ratio. Different letters within column indicate significant differences in means (*p* <0.05).

The FTIR spectra of all FRNs are shown in Fig. 9. As shown in Fig. 9(a), no new chemical bonds or functional groups were generated in the FTIR spectra of FRNs with BTWE addition as compared to the control group. This indicates that no new groups were formed between the tea polyphenols and starch molecules due to chemical reactions, and that the interaction forces between the former two substances were non-covalent, such as hydrogen bonding, electrostatic forces, and hydrophobic interactions (He et al., 2021). The peaks formed within the wavelength range of 3200–3600 cm^−1^ corresponded to the intermolecular hydrogen bonded and O—H stretching vibration associated with bound water and starch, and N—H bond stretching vibrations related to proteins (Różyło et al., 2023). As shown in Fig. 9(b), the characteristic peak at 3400 cm^−1^ in the FTIR spectra of FRNs with BTWE was broadened with increasing tea:water ratio as compared to that of the control group, indicating that BTWE addition promoted the generation of intermolecular stronger hydrogen bonds between molecules (Niu et al., 2018). Therefore, it can be surmised that tea polyphenols may interact with starch to form hydrogen bonds, thereby reducing the free starch content and impeding the development of an ordered structure (Lv, Li, et al., 2020a), which is also consistent with previous XRD results.

Moreover, in the FTIR spectra, the ratio of R_1047/1022_ can be used as an indicator for characterizing the short-range ordered structure of starch molecules. The larger the ratio, the higher is the short-range order and the larger is the crystalline region of the starch (Ding et al., 2019). As shown in Fig. 9(c), the R_1047/1022_ ratios of FRNs treated with BTWE decreased compared to the control group, with significant reductions observed in both the control group and other groups with tea:water ratios of 8:100 and 10:100. This indicated that polyphenols disrupted the primitive intermolecular hydrogen bonding and intermolecular dissociation of starch molecular chains through interactions with starch. This gradually weakened the structural characteristics of the crystalline region of the starch granules into amorphous forms and affected the formation of relatively ordered structures (Zheng et al., 2020). Furthermore, typical characteristic peaks at the range of 1700–1520 cm^−1^ were attributed to stretching vibrations of the C=O bond (amide I bond), bending vibration of N—H bond, and the stretching vibration of C—N double bond stretch-coupling bond (amide II bond) (Li, Xiao, et al., 2021). The deconvolution results of the amide I band (1700–1580 cm^−1^) are shown in Fig. 9(d), which indicated the presence of α-helix secondary structures of proteins in FRNs. As shown, the reduction in α-helix content indicated disruption of the surface hydrogen bond network, resulting in destabilization of the protein secondary structure. In combination with Fig. 9(a), it could be seen that FRNs with tea:water ratios of 4:100, 6:100, and 8:100 showed a significant decrease in the intensity of this band compared to the control group, which might be attributed to the weakened hydrogen bounds between protein peptide chains caused by starch-protein interactions (Shi et al., 2020). This indicates that the proteins actively participated in the reaction, playing a crucial role in the structure remodeling of FRNs.

## 4 Conclusion

This study investigated the effects of different BTWE concentrations on the quality characteristics and microstructure of FRNs. The results showed that the addition of BTWE resulted in a richer color, enhanced flavor, and significantly increased antioxidant capacity of FRNs. When BTWE was incorporated at low-concentration (ranging from 4:100 to 8:100), its phenolic compounds interacted with starch and protein molecules, facilitating the formation of a more robust three-dimensional gel network. This structural enhancement resulted in a denser gel structure with reduced pore size, significantly improving the structural properties, and reducing the cooking loss of FRNs. Furthermore, the molecular mechanism revealed that competitive hydrogen-bonding interactions between high-concentration tea polyphenols and starch molecules inhibited the formation of starch intermolecular hydrogen bonds, thereby affecting the formation of molecularly ordered structures. In conclusion, low-concentrations of BTWE can effectively enhance the structural properties of FRNs, as phenolic compounds interact with starch and protein, promoting a more robust three-dimensional gel network.

However, BTWE is a complex mixture of various components, and its functional active ingredients have not been isolated and evaluated. While this study comprehensively elucidates the mechanism by which BTWE improves the quality of FRNs, the specific roles and contributions of individual components or compound classes remain unclear. Therefore, future research will focus on isolating and identifying the primary active ingredients in BTWE, as well as systematically investigating their interactions with rice noodle components and specific effects on quality enhancement. These efforts aim to establish a solid theoretical foundation and provide technical support for the development of functional, health-oriented rice noodle products. Furthermore, to assess the generalizability of tea extracts as natural food fortifiers, further studies should investigate the fundamental impact of raw material variation on the overall quality enhancement of tea-enriched rice noodles by incorporating a broader range of tea varieties.

## CRediT authorship contribution statement

**Tong Chen:** Writing – original draft, Software, Data curation. **Rongrong Xu:** Writing – original draft, Visualization, Validation, Software. **Xiaohui Gao:** Software, Methodology. **Shuyu Wei:** Writing – review & editing, Supervision. **Qianwei Cheng:** Writing – review & editing, Supervision, Resources, Methodology. **Luli Meng:** Software, Resources. **Jiayan Zhang:** Supervision. **Yuan Cheng:** Writing – review & editing, Resources, Methodology, Conceptualization.

## Declaration of competing interest

The authors declare that they have no known competing financial interests or personal relationships that could have appeared to influence the work reported in this paper.

## Data Availability

Data will be made available on request.
